# Predicting late anemia in critical illness

**DOI:** 10.1186/cc4847

**Published:** 2006-02-28

**Authors:** Eric B Milbrandt, Gilles Clermont, Javier Martinez, Alex Kersten, Malik T Rahim, Derek C Angus

**Affiliations:** 1 The CRISMA Laboratory (Clinical Research, Investigation, and Systems Modeling of Acute Illness), Department of Critical Care Medicine, University of Pittsburgh School of Medicine, 3550 Terrace Street, Pittsburgh, PA 15261, USA

## Abstract

**Introduction:**

Identifying critically ill patients most likely to benefit from pre-emptive therapies will become increasingly important if therapies are to be used safely and cost-effectively. We sought to determine whether a predictive model could be constructed that would serve as a useful decision support tool for the pre-emptive management of intensive care unit (ICU)-related anemia.

**Methods:**

Our cohort consisted of all ICU patients (*n *= 5,170) admitted to a large tertiary-care academic medical center during the period from 1 July 2000 to 30 June 2001. We divided the cohort into development (*n *= 3,619) and validation (*n *= 1,551) sets. Using a set of demographic and physiologic variables available within six hours of ICU admission, we developed models to predict patients who either received late transfusion or developed late anemia. We then constructed a point system to quantify, within six hours of ICU admission, the likelihood of developing late anemia.

**Results:**

Models showed good discrimination with receiver operating characteristic curve areas ranging from 0.72 to 0.77, although predicting late transfusion was consistently less accurate than predicting late anemia. A five-item point system predicted likelihood of late anemia as well as existing clinical trial inclusion criteria but resulted in pre-emptive intervention more than two days earlier.

**Conclusion:**

A rule-based decision support tool using information available within six hours of ICU admission may lead to earlier and more appropriate use of blood-sparing strategies.

## Introduction

Most intensive care therapies are prescribed in response to the development of organ dysfunction or other unwanted events. For example, antibiotics are prescribed when a patient develops sepsis, or tidal volumes are lowered when a patient develops acute respiratory distress syndrome (ARDS) [1]. In other arenas of medicine, therapies are much more commonly provided to populations who are at risk of unwanted events, in the hope of future benefit. For example, antihypertensives and lipid-lowering agents are given to prevent cardiovascular disease long before any hint of the disease has ever manifested. It is likely that similar pre-emptive strategies will reap gains in the intensive care unit (ICU). For example, it seems that use of low tidal volumes in patients at risk of ARDS reduces the likelihood of their subsequently developing ARDS [2]. However, critically ill patients are fragile and complex, so broad use of early therapies with a theoretical hope of avoiding or mitigating downstream consequences will probably need to be balanced against the costs and risks of the therapies themselves. Thus, strategies to identify the candidates most likely to benefit will become increasingly important if therapies are to be used safely and cost-effectively.

In this article we chose anemia of critical illness as an illustrative example of a condition that one might want to manage pre-emptively. Trials are currently ongoing to assess whether treatment with recombinant human erythropoietin (rHuEPO) can mitigate anemia-associated morbidity or mortality. If the outcome of these trials is positive, we will be faced with an interesting challenge. Specifically, we will have a therapy that is expensive, associated with small but potentially important side effects, unlikely to offer any benefit to patients who have short ICU courses and are at no risk of anemia of critical illness, and yet only able to produce significant improvements in serum hemoglobin levels after several days of treatment. Therefore, to be most effective it must be given early in the ICU course, and to be most efficient it must be given only to the subset of ICU patients who will end up incurring a prolonged hospital stay and developing anemia. The dilemma of finding the right subset of patients before they have developed the outcome of interest is a classic decision-making problem, with similarities to other situations that may soon arise in critical care, such as early tracheostomy [3] or the prophylactic use of antidepressants [4].

The goal of this paper was to determine whether a predictive model could be constructed that would serve as a useful decision support tool for the pre-emptive management of ICU-related anemia. We stress that this exercise is only theoretical at this point, because there is insufficient evidence to recommend rHuEPO as therapy for anemia of critical illness. However, we believe the exercise sheds light on the potential opportunities for similar models for other pre-emptive management strategies in the ICU.

## Materials and methods

Our approach was to build and validate a model-based rule that used information available within six hours of ICU admission to predict the likelihood that a patient would have a prolonged hospital course complicated by anemia. We then compared the potential consequences of a prescription strategy that relied on the rule with those of a strategy that uses the existing clinical trial inclusion criteria [5] and with those of a broad-based ICU prescription strategy.

### Patients and data sources

For our study cohort we selected all ICU patients admitted during the period from 1 July 2000 to 30 June 2001 at the University of Pittsburgh Medical Center, a hospital with more than 120 ICU beds caring for adult medical, surgical, trauma, neurologic, and solid-organ transplant patients. We obtained information from several computerized databases, including administrative discharge data, laboratory data, itemized billing records, and a detailed bedside database containing the following prospectively collected data for all ICU patients: demographics, admission diagnosis, physiologic data, and interventions (medications, fluids and blood products, mechanical ventilation, and renal replacement). To preserve patient confidentiality, all data were de-identified and linked by an honest broker. The data were cleaned (purged of inconsistent and/or nonsense values), organized, and merged to create files for the analysis. We then generated composite variables, such as the presence of organ dysfunction, from detailed physiologic data. Data were manipulated as a relational database in Microsoft FoxPro 6.0. The University of Pittsburgh Institutional Review Board approved the study.

### Modeling and statistical analyses

The cohort was divided into development (two-thirds of cohort) and validation (one-third of cohort) sets. Using a set of demographic and physiologic variables available within six hours of ICU admission, we developed two predictive models using logistic regression. The first model was developed to identify patients who received 'late transfusion', namely those who received red cell transfusion(s) 7 days or more after ICU admission. The second model was developed to identify patients who developed 'late anemia', namely those whose actual or predicted hemoglobin reached 7 g/dl or less at seven days or more after ICU admission. The threshold of 7 g/dl was based on the randomized controlled trial by Hebert and colleagues [6], which found a lower hospital mortality for subjects transfused below this value compared with a more liberal (10 g/dl) transfusion threshold. For patients with pre-transfusion hemoglobin concentrations of more than 7 g/dl, we extrapolated these values to determine who would have reached 7 g/dl by using the mean of individual slopes of hemoglobin concentrations. Individual slopes were constructed from the calculated day-to-day decrease in hemoglobin in patients not receiving transfusions [7].

Independent predictors eligible for inclusion in the two models were as follows: age (less than 65 years, 65 years or more), gender, race (white, black or other), comorbidity [8], body mass index, medical or surgical admission, emergency surgery, trauma, and the following data obtained within six hours of ICU admission: minimum hemoglobin, red cell transfusion (yes/no), creatinine (less than 1.6 mg/dl, 1.6 mg/dl or more), international normalized ratio (1.9 or less, more than 1.9), arterial blood lactate (1.5 mg/dl or less, more than 1.5 mg/dl), and need for mechanical ventilation or vasopressors. Model discrimination and calibration were tested by using the receiver operator characteristic (ROC) curve area and the Hosmer–Lemeshow test. In secondary analyses, we developed models using data available within 24, 48, and 72 hours of ICU admission. Using the 6-hour model for late anemia, we constructed a point system to quantify, on the first ICU day, the likelihood of their reaching a hemoglobin value of 7 g/dl or less at 7 days or more after ICU admission and therefore being a suitable candidate for a blood-sparing strategy such as rHuEPO. We then compared the potential consequences of a prescription strategy that relied on the rule with those of a strategy that uses the existing clinical trial inclusion criteria (ICU length of stay 3 days or more, age 18 years or more, and hematocrit less than 38%) [5] and with those of a broad-based ICU prescription strategy. All data analyses were performed and models developed with SPSS 11.5 (Chicago, IL, USA). We assumed statistical significance for differences between groups or model coefficients at *p *< 0.05.

## Results

### Patient characteristics

**Figure 1:**
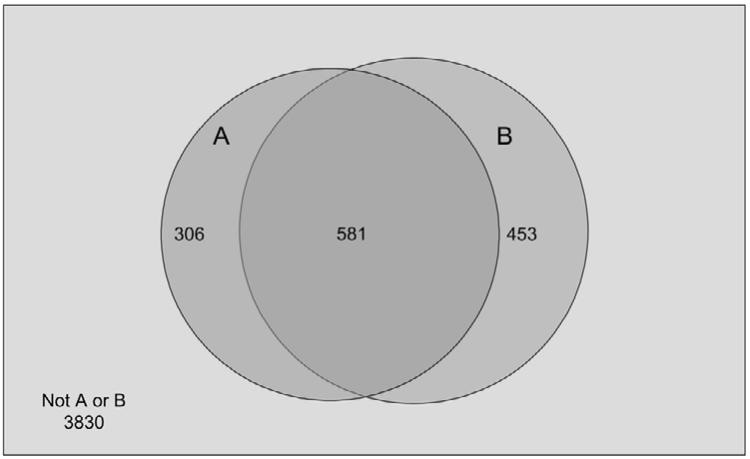
Venn diagram of patient groups. More patients met criteria for late anemia (group B, *n *= 1,034) than for late transfusion (group A, *n *= 887). More than one-quarter (1,340/5,170; 25.9%) of all patients admitted to the intensive care unit met criteria for at least one group. Although there is considerable overlap, a significant proportion of patients (759/5,170; 14.7%) met only one set of criteria.

A total of 5,170 ICU patients were admitted from 1 July 2000 to 30 June 2001 (Table 1), with 887 (17.2%) and 1,034 (20.0%) in the late transfusion and late anemia groups, respectively. There was a fair degree of overlap between the two groups (Figure 1). Within six hours of ICU admission, patients in either group were significantly more likely than those who did not belong to each group to have at least one comorbidity, lower body mass index, surgical admission type, transfusion, mechanical ventilation, inotrope use, and higher creatine, international normalized ratio, and lactate (all *p *< 0.05). In addition, those in the late transfusion group were less likely to have undergone emergent surgery, whereas those in the late anemia had lower initial hemoglobin values and were more likely to have been less than 65 years of age and admitted for trauma.

### Model development and validation

**Figure 2:**
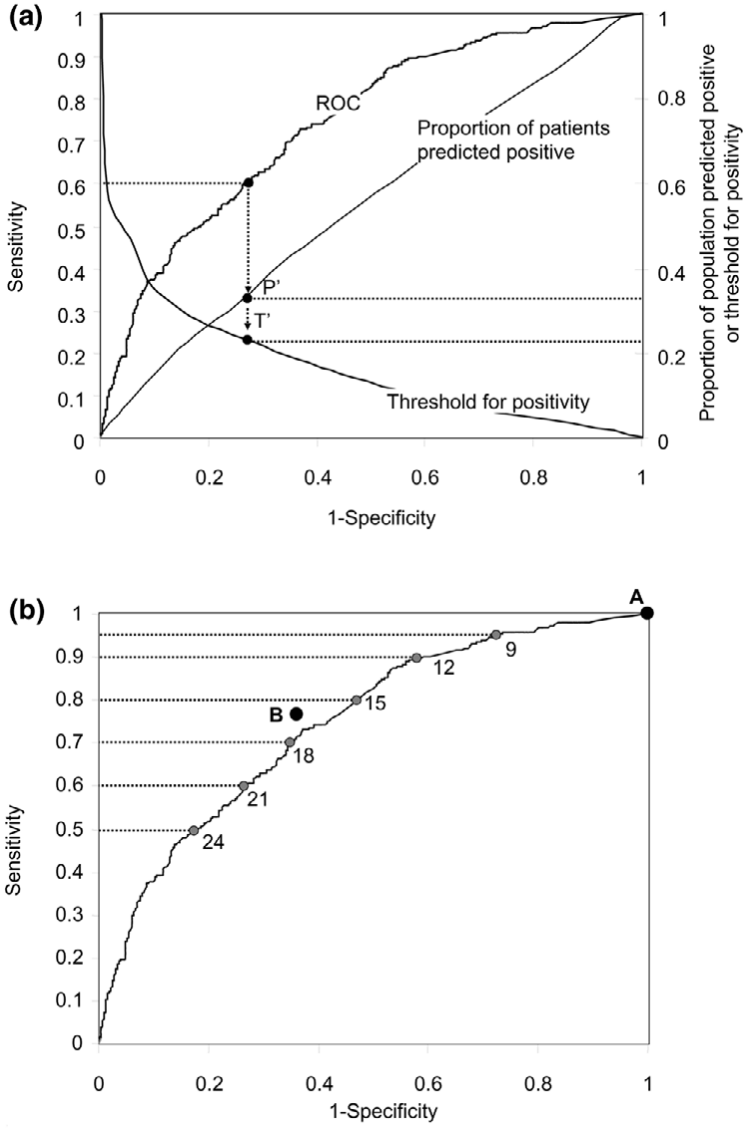
Receiver operating characteristics of late anemia prediction model and correspondence with point system. **(a) **The receiver operating characteristic (ROC) of the model predicting late anemia. Higher sensitivity (S) is associated with a lower threshold for positivity (T', probability of meeting criteria for late anemia). Accordingly, a higher proportion of the population will be declared to meet late anemia criteria (P') as specificity decreases. For example, a desired sensitivity of 60% would entail declaring a threshold for positivity corresponding to a probability of late anemia of 22% or more (T'). This results in 34% of the population exceeding this predicted probability and thus at risk for late anemia (P'). **(b) **Correspondence between intensive care unit (ICU) anemia points (see the text) and the ROC curve. Determining a lower point threshold of unacceptable risk of late anemia entails a trade-off between higher sensitivity and the willingness to declare a higher proportion of the population at risk. Setting a threshold of 21 points, which would achieve a sensitivity of 60% in identifying patients meeting criteria for late anemia, would result in considering intervention in 34% of the population (a). On the assumption that the proposed intervention is recombinant human erythropoietin, considering all ICU patients for treatment would result in 100% sensitivity, but no specificity (point A). Using inclusion criteria for the Corwin study results in 77% sensitivity and 65% specificity as illustrated by point B (see the text and Table 4), which is comparable to the point-based system. The ICU anemia score permits earlier intervention with comparable predictive power.

The population was divided into development (*n *= 3,619) and validation (*n *= 1,551) cohorts. Independent predictors for late transfusions and late anemia are provided in Table 2. The ROC curve areas and Hosmer–Lemeshow test *p *values for the development and validation cohorts for late transfusion were 0.75 (*p *= 0.06) and 0.72 (*p *= 0.46), respectively, and the corresponding values for late anemia were 0.77 (*p *= 0.01) and 0.74 (*p *= 0.05). The ROC curve for the development cohort of the late anemia model is depicted in Figure 2a. This figure also illustrates the correspondence between sensitivity and the fraction of the study population that exceeds the positivity threshold and is therefore considered at risk for late anemia for a given sensitivity level. Higher sensitivity results in lower specificity, corresponding to a higher proportion of the population being considered at risk. For example, a desired sensitivity of 60% entails declaring a threshold for positivity corresponding to a probability of late anemia of 22% or more (T'), resulting in 34% of the population exceeding this predicted probability and thus being considered at risk for late anemia (P') and potential candidates for intervention. For a desired sensitivity of 90%, the corresponding probability of late anemia would be 10% or more, resulting in the judging of 64% of the population to be at risk. Higher discrimination; in other words a greater ROC curve area, would allow a smaller proportion of the population to be considered at risk for a given sensitivity. In secondary analyses we constructed models that included the same risk factors but evaluated within 24, 48, and 72 hours of ICU admission. These models showed modest improvements in discrimination (Table 3), yet they resulted in progressively greater delays in identifying patients at risk of late transfusion or late anemia. It is noteworthy that predicting late transfusion was consistently less accurate than predicting late anemia.

### Development of an ICU anemia score

**Figure 3:**
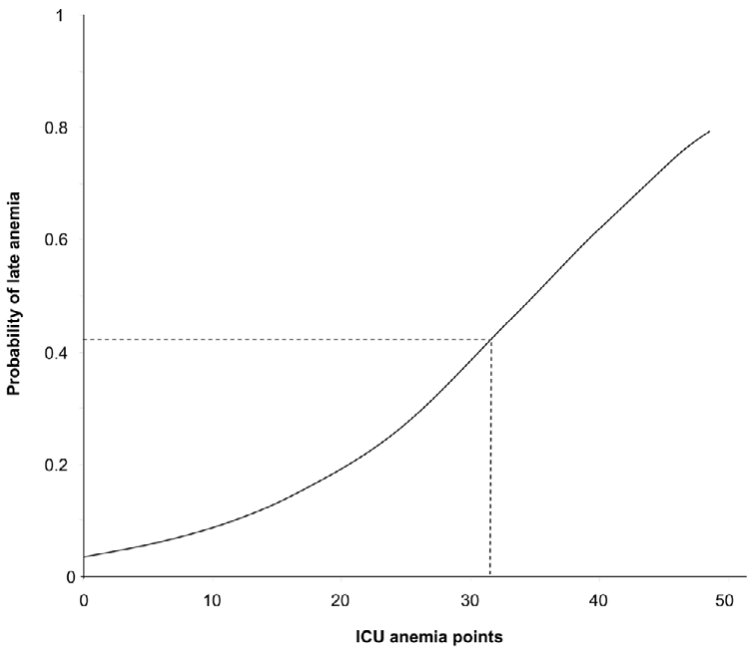
Intensive care unit anemia point system. The correspondence between intensive care unit anemia points (see the text and Table 4) and probability for meeting criteria for late anemia is shown. For example, a surgical patient (11 points) with a lactate concentration of 4.0 g/dl (6 points) and a hemoglobin level of 9 g/dl (15 points) acquires 32 points, which corresponds to a risk of late anemia of 42.4%. The maximum score does not correspond to a probability of 1.00 because patients dying in the first week will never meet late anemia criteria.

We wished to improve the practicality and possible clinical utility of a prediction rule for patients at risk for late anemia who might benefit from a blood-sparing strategy. Using the late anemia model we constructed a point system to calculate, within 6 hours of ICU admission, the likelihood of reaching the outcome of interest, a hemoglobin value of 7 g/dl or less at 7 days or more after ICU admission. We assumed that a baseline hemoglobin of 14 g/dl or more and the absence of any risk predictors would amount to a minimum score of zero and thus a minimum chance for reaching the outcome. The maximal score of 51 corresponds to a baseline hemoglobin of 7 and the presence of all risk factors. Interestingly, this maximal score corresponds to a predicted probability of outcome of 0.81 as opposed to 1.00, reflecting that patients with an excessive burden are likely to die before 7 days. The ICU anemia point scheme, described in Table 4, demonstrated good discrimination, with an ROC curve area of 0.74. The correspondence between the sensitivity of the model in identifying patients that suffered late anemia and the corresponding number of points is shown in Figures 2b and 3.

Recommending a specific threshold beyond which patients should be considered at risk for late anemia and thus as candidates for a blood-sparing strategy depends on the cost and possible side effects of this strategy. Willingness to broaden the criteria to implement the strategy is more forthcoming with a low risk/low cost strategy. From Figure 2b, one might consider as candidates patients with ICU anemia scores in excess of 12 (more than 90% sensitivity; 64% of the population are candidates). Costlier or riskier strategies would dictate higher thresholds (for example an ICU anemia score of more than 21; more than 60% sensitivity; 34% of the population are candidates).

### Potential consequences of point-based system and other prescriptive strategies

On the assumption that the proposed intervention is rHuEPO, treatment of all comers regardless of baseline or clinical characteristics resulted in 100% of patients who needed the intervention getting it, but also 100% of patients who did not (Table 5 and Figure 2b, point A). Application of the Corwin trial inclusion criteria [5] to patients in our cohort correctly targeted for treatment 77.4% (800/1,034) of those who were destined to develop late anemia (Table 5). However, it also resulted in the treatment of 35.5% (1,470/4,136) of patients who did not develop late anemia. When plotted against the ICU anemia score ROC curve, the prediction offered by the Corwin criteria (Figure 2b, point B) falls near the curve, indicating that the Corwin criteria perform as well as the point-based system. However, the Corwin criteria require a patient to be in the ICU for 3 days before initiating treatment, resulting in a delay of more than 2 days in treatment initiation in comparison with the point-based system, which depends only on data available within 6 hours of ICU admission.

## Discussion

Using information available within six hours of ICU admission, we were able predict the likelihood of late anemia with reasonable accuracy, suggesting a window of opportunity for the early identification of patients who might benefit from transfusion-sparing strategies. Additionally, the application of predictors in the form of a rule-based decision support tool resulted in a much larger proportion of patients 'appropriately' receiving intervention as opposed to more broad-based use and earlier intervention than with existing trial inclusion criteria [5]. Although this exercise was theoretical, it has the potential to increase the safe and cost-effective use of rHuEPO and other transfusion-sparing strategies. Furthermore, we believe it is illustrative of the potential opportunities for similar models for other pre-emptive management strategies in the ICU.

The identification of candidates most likely to benefit from an early intervention targeting late sequelae involves a trade-off between accuracy and timeliness. In our models, including risk factors from within 24, 48, or 72 hours of ICU admission, as opposed to only the first 6 hours, improved discrimination, but at a cost of a delays in starting the intervention. Similarly, using existing clinical trial inclusion criteria to select patients for intervention led to a delay of more than 2 days in treatment. For an intervention such as rHuEPO, which takes days to begin to show an effect, a delay of 1 or 2 days may be unacceptable. For less time-sensitive pre-emptive strategies, such delays may not be an issue. The importance of timeliness must be balanced with that of accuracy, which becomes more important as th erisk and cost of a particular intervention increase. Clearly, identification strategies will need to be tailored for each intervention.

Predicting late transfusion was consistently less accurate than predicting late anemia. Given the substantial variation in transfusion practices [9] it should not be surprising that predicting transfusion was difficult. As transfusion practices become more standardized and restrictive transfusion thresholds the norm, the ability to predict transfusion is likely to become easier. However, as we have witnessed at our own institution, even with strict enforcement of transfusion thresholds, there are still many apparently unjustified transfusions that continue to add unnecessary variation.

There are several limitations that deserve consideration. Because of the retrospective nature of this study, we were not able to determine why patients received blood transfusion. Because so few hemoglobin concentrations ever fell to 7 g/dl before transfusion, we had to extrapolate pre-transfusion hemoglobin values to determine who would have reached 7 g/dl by using the mean of individual slopes of hemoglobin concentrations. Although it might have been more accurate to develop a predictive model using a large group of patients whose hemoglobin concentrations actually fell to the threshold, such a group does not exist, even among the 5,170 ICU patients in our study. Importantly, the mean hemoglobin slope after the acute phase (more than 3 days) used for this extrapolation (about 0.22 g/dl per day) was consistent with what others have reported previously [7] and are therefore likely to be a reasonable approximation. To develop our models we relied on information obtained from several computerized databases, an approach that would miss potential predictors that were not recorded in these databases. This was a single-center study at a tertiary-care academic medical center. It is possible that predictors of late anemia at this institution might vary from those of other institutions, especially those with significantly different case-mix or ICU practices. In light of this and because this exercise was only intended to be theoretical at this point, the ICU anemia point system should be validated in other cohorts before being applied to routine patient care.

## Conclusion

A rule-based decision support tool using information available within six hours of ICU admission could lead to more appropriate and timely use of transfusion-sparing interventions. In the future, strategies to identify critically ill patients most likely to benefit from pre-emptive therapies will become increasingly important if therapies are to be used safely and cost-effectively.

## Key messages

• 	Identifying critically ill patients most likely to benefit from pre-emptive therapies will become increasingly important if therapies are to be used safely and cost-effectively.

• 	By using information available within 6 hours of ICU admission, we were able predict the likelihood of late anemia with reasonable accuracy, suggesting a window of opportunity for the early identification of patients who might benefit from transfusion-sparing strategies.

• 	The application of predictors in the form of a rule-based decision support tool resulted in a much larger proportion of patients 'appropriately' selected for intervention.

• 	A rule-based method has the potential to increase the safe and cost-effective use of rHuEPO and other transfusion-sparing strategies in critically ill patients admitted to the ICU.

## Abbreviations

ARDS = acute respiratory distress syndrome; ICU = intensive care unit; rHuEPO = recombinant human erythropoietin; ROC = receiver operating characteristic.

## Competing interests

This work was supported by a research grant from Amgen, Inc. The authors were solely responsible for study conceptualization, analysis, and manuscript preparation without input or oversight from the sponsor.

## Authors' contributions

EBM participated in the design of the study, acquisition, analysis and interpretation of the data, and drafted the manuscript. GC participated in the design of the study, acquisition, analysis and interpretation of the data, and helped draft the manuscript. JM, AK, and MTR participated in the acquisition, analysis and interpretation of the data and helped draft the manuscript. DCA conceived of the study, participated in its design and coordination, and helped draft the manuscript. All authors read and approved the final manuscript.

## Figures and Tables

**Table 1:** Demographic and clinical variables

Variable	All patients	Late transfusion	No late transfusion	Late anemia	No late anemia
Subjects (*n*)	5,170	887	4,283	1,034	4,136
Age, years (mean ± SD; median)	60.5 ± 17.4; 62.5	60.9 ± 17.4; 62.2	60.4 ± 17.4; 62.6	60.1 ± 17.2; 61.5	60.6 ± 17.5; 62.8
Age 65 years or more (%)	47.1	46.1	47.3	44.3*	47.8
Race (%)					
White	85.3	85.3	85.2	85.6	85.2
Black	8.4	8.3	8.5	8.8	8.3
Others and unknown	6.3	6.3	6.3	5.6	6.5
Gender (percentage females)	43.6	44.5	43.5	44.6	43.4
Co-morbidity^a ^(%)	51.5	57.7^c^	50.3	57.3^c^	50.1
Diabetes	17.5	17.5	17.5	17.2	17.5
Pulmonary disease	9.8	12.3^c^	9.3	10.7	9.6
Renal disease	0.2	0.8^c^	0.1	0.4	0.2
Liver disease	7.3	14.3^c^	5.9	13.8^c^	5.7
Peripheral vascular disease	5.0	6.5^c^	4.6	4.7	5.0
Cerebrovascular disease	1.8	1.8	1.8	2.1	1.7
Myocardial infarction	7.2	4.5^c^	7.7	5.7^c^	7.5
BMI (mean ± SD; median)	27.7 ± 7.4; 26.5	27.0^c ^± 7.3; 25.7	27.9 ± 7.4; 26.6	27.3^c ^± 7.1; 25.9	27.9 ± 7.4; 26.6
Medical (%)	41.2	21.1^c^	45.4	19.2^c^	46.7
Emergency surgery (%)	17.4	15.1^c^	17.9	17.4	17.4
Trauma case (%)	12.0	13.5	11.7	14.9^c^	11.2
Admission diagnosis^b ^(%)					
Medical					
Cardiovascular	3.9	3.6	3.9	3.6	3.9
Respiratory	7.1	7.8	6.9	5.5^c^	7.5
Gastrointestinal	5.5	6.4	5.3	7.5^c^	5.0
Neurologic	7.8	6.5	8.1	4.8^c^	8.6
Sepsis	2.4	4.5^c^	2.0	3.5^c^	2.2
Nonoperative trauma	4.9	6.1	4.6	5.0	4.9
Metabolic	2.1	0.9^c^	2.3	0.8^c^	2.4
Renal	0.3	0.2	0.3	0.3	0.3
Other medical	0.9	0.9	0.8	0.6	0.9
Surgical					
Cardiovascular	17.8	8.5^c^	19.8	13.0^c^	19.1
Respiratory	1.9	1.8	1.9	2.0	1.9
Gastrointestinal	3.7	4.5	3.5	4.6	3.5
Neurologic	3.1	2.6	3.2	3.5	3.0
Operative trauma	2.7	4.1^c^	2.4	5.4^c^	2.0
Other surgical	1.1	1.2	1.1	1.6	1.0
Unknown	34.9	40.4^c^	33.7	38.2^c^	34.0
APACHE III score (mean ± SD; median)	48.2 ± 25.1; 44.0	56.4^c ^± 23.9; 54.0	46.7 ± 25.0; 42.0	54.9^c ^± 23.6; 51.0	46.7 ± 25.1; 42.0
MV at any time (%)	30.6	56.0^c^	25.4	50.4^c^	25.7
ICU LOS, days (mean ± SD; median)	6.3 ± 9.7; 3.0	17.8^c ^± 16.9; 14.0	3.9 ± 4.7; 2.0	15.5^c ^± 16.2; 11.0	4.0 ± 5.2; 2.0
Hospital LOS days (mean ± SD; median)	15.0 ± 16.3; 9.0	33.8^c ^± 24.0; 27.0	11.1 ± 10.7; 8.0	30.0^c ^± 22.7; 24.0	11.2 ± 11.6; 8.0
Hospital mortality (%)	12.9	20.3^c^	11.4	13.5	12.7
Physiologic and therapeutic measures within 6 hours of ICU admission					
Hemoglobin, g/dl (mean ± SD; median)	9.6 ± 4.7; 10.7	9.5 ± 3.6; 10.0	9.6 ± 4.9; 10.9	9.1^c ^± 3.6; 9.7	9.7 ± 4.9; 11.1
Lactate more than 1.5 mg/dl (%)	14.7	26.5^c^	7.7	26.3^c^	11.8
Creatinine at least 1.6 mg/dl (%)	15.7	26.0^c^	13.5	22.7^c^	13.9
INR more than 1.9 (%)	5.7	10.1^c^	4.8	10.0^c^	4.7
Inotropes (%)	10.6	17.7^c^	9.1	17.3^c^	8.9
Mechanical ventilation (%)	16.3	23.9^c^	14.8	22.2^c^	14.8
Transfusion (%)	8.3	11.2^c^	7.7	17.2^c^	6.1

Late transfusion, transfused 7 days or more after admission to intensive care unit; late anemia, reached or would have reached 7.0 g/dl at 7 days or more after admission to intensive care unit. APACHE, Acute Physiology and Chronic Health Evaluation; BMI, body mass index; ICU, intensive care unit; LOS, length of stay; MV, mechanical ventilation; INR, international normalized ratio. ^a^Any co-morbidity, as measured in accordance with Charlson–Deyo procedures for the index admission with International Classification of Diseases, Ninth Revision, Clinical Modification (ICD-9-CM) discharge diagnoses. ^b^Admission diagnosis, which represents the condition most likely to be responsible for ICU admission as reported in the electronic medical record, was not available for 35% of patients. Because of rounding, totals may not equal 100%. ^c^*p *< 0.05 between patients belonging to group of interest and those who did not belong to this group.

**Table 2:** Risk factors for late transfusion or late anemia from multivariable models

Risk factor^a^	Odds ratio (95% confidence interval)	
	Late transfusion	Late anemia
Abnormal lactate (> 1.5 mg/dl)	1.64 (1.28–2.09)	1.79 (1.41–2.26)
Transfused	0.64 (0.45–0.87)	-
Surgical patient	2.51 (1.86–3.40)	2.90 (2.19–3.83)
Non-emergent surgery	1.72 (1.22–2.22)	1.60 (1.26–2.04)
Mechanical ventilation	1.73 (1.37–2.19)	-
Hemoglobin deficit^b^	1.21 (1.16–1.26)	1.30 (1.25–1.35)
Abnormal creatinine (≥ 1.6 mg/dl)	1.75 (1.38–2.21)	-
Inotropic support	-	1.69 (1.29–2.21)

Late transfusion, transfused 7 days or more after admission to intensive care unit; late anemia, reached or would have reached 7.0 g/dl at 7 days or more after admission to intensive care unit. ^a^Worst value recorded up to 6 hours after admission to intensive care unit. ^b^Calculated as g/dl lower than 14 g/dl, with zero deficit defined as a baseline hemoglobin of at least 14 g/dl.

**Table 3:** Receiver operator characteristics of predictive models

Model	Receiver operator characteristic curve area			
	Late transfusion		Late anemia	
	Development	Validation	Development	Validation
6-hour	0.75	0.72	0.77	0.74
24-hour	0.75	0.73	0.81	0.78
48-hour	0.76	0.74	0.84	0.82
72-hour	0.77	0.75	0.86	0.86

Late transfusion, transfused 7 days or more after admission to intensive care unit; late anemia, reached or would have reached 7.0 g/dl at 7 days or more after admission to intensive care unit.

**Table 4:** The intensive care unit anemia score

Risk factor	Points^c^
Abnormal lactate^a ^(> 1.5 mg/dl)	6
Inotropic support	5
Surgical patient	11
Non-emergent surgery	5
Each g/dl of hemoglobin less than 14 g/dl^a,b^	3

^a^Calculated as the worst value at 6 hours after admission to intensive care unit. ^b^If baseline hemoglobin is 14 g/dl or more, the deficit is 0. ^c^For example, a surgical patient (11 points) with a lactate concentration of 4.0 g/dl (6 points) and a hemoglobin level of 9 g/dl (15 points) earns 32 points. From Figure 3, this corresponds to a risk of late anemia of 42.4%.

**Table 5:** Potential consequences of alternative prescriptive strategies

Strategy	Number not developing late anemia (*n *= 4,136)		Number developing late anemia (*n *= 1,034)		Predictive strength			
	Not treated	Treated	Not treated	Treated	Sensitivity	Specificity	PPV	NPV
Corwin^a^	2,666	1,470	234	800	0.77	0.65	0.35	0.92
Broad-based^b^	0	4,136	0	1,034	1.00	0	0.20	Undefined

Late anemia, reached or would have reached 7.0 g/dl at 7 days or more after admission to intensive care unit. NPV, negative predictive value; PPV, positive predictive value. ^a^Patients were treated on the basis of clinical trial inclusion criteria in reference [5]. ^b^Strategy in which all patients are treated.
